# Googling Insomnia, Light, Metabolism, and Circadian: A Population Interest Simple Report

**DOI:** 10.3390/brainsci12121683

**Published:** 2022-12-08

**Authors:** Emanuele Di Simone, Nicolò Panattoni, Alfredo De Giorgi, Pedro Manuel Rodríguez-Muñoz, Marta Bondanelli, Francisco José Rodríguez-Cortés, Pablo Jesús López-Soto, Noemi Giannetta, Sara Dionisi, Marco Di Muzio, Fabio Fabbian

**Affiliations:** 1 Nursing, Technical, Rehabilitation, Assistance and Research Direction-IRCCS Istituti Fisioterapici Ospitalieri-IFO, 00144 Rome, Italy; 2 Clinica Medica Unit, University Hospital of Ferrara, 44124 Ferrara, Italy; 3 Department of Nursing and Physiotherapy, Universidad de Salamanca, 37008 Salamanca, Spain; 4 Department of Nursing, Instituto Maimónides de Investigación Biomédica de Córdoba, 14004 Córdoba, Spain; 5 Department of Medical Sciences, University of Ferrara, 44121 Ferrara, Italy; 6 Department of Nursing, Pharmacology and Physiotherapy, Universidad de Córdoba, 14004 Córdoba, Spain; 7 Hospital Universitario Reina Sofía de Córdoba, 14004 Córdoba, Spain; 8 School of Nursing, UniCamillus-Saint Camillus International University of Health and Medical Sciences, 00131 Rome, Italy; 9 Department of Clinical and Molecular Medicine, Sapienza University of Rome, 00185 Rome, Italy

**Keywords:** Google Trend^®^, insomnia, light, metabolism, circadian, infodemiology

## Abstract

Exposure to light at night, insomnia, and disrupted circadian patterns could be considered risk factors for developing noncommunicable diseases. Understanding the awareness of the general population about the abovementioned factors could be essential to predict noncommunicable diseases. This report aimed to investigate the general community’s interest in circadian, insomnia, metabolism, and light using Google Trends, and to evaluate results from different geographic areas. Relative search volumes (RSVs) for the factors mentioned, filtered by the “Health” category, were collected between 2007 and 2021. Moreover, RSVs were analysed in five different European languages. Worldwide mean RSVs for “Circadian”, “Insomnia”, “Light”, and “Metabolism” during the study period were 2%, 13.4%, 62.2%, and 10%, respectively. In different developed countries, searching for light, insomnia, and metabolism were different, suggesting a variable level of awareness. Limited knowledge about the circadian pattern of human activities was detected. The highest correlation coefficient was calculated. Our results suggest the potential role of extensive data analysis in understanding the public interest and awareness about these risk factors. Moreover, it should be interpreted as the onset of stimulus for researchers to use comprehensible language for reaching comprehensive media coverage to prevent sleep and circadian system disturbances.

## 1. Introduction

The expansion of light-emitting diode (LED) lighting at both the environmental and domestic level, and consequent exposure to artificial light at night (ALAN), plays a disrupting effect on the biological clock and affects sleep and health [1,2]. Although we do not yet know all the functions of sleep, we spend roughly one-third of our life sleeping. It is estimated that only two-thirds of Americans each night can sleep the recommended 7 h [3]. Ohayon and Milesi [4] performed an observational study on more than 19,000 adult subjects living in 15 US states based on satellite recording, geocoding addresses, and phone interviews. The results showed that living in areas with greater outdoor night-time light was significantly associated with delayed bedtime and wake-up time, shorter sleep duration, and increased daytime sleepiness. Health status is affected by shorter sleep duration and light exposure, with sleep deprivation being strongly connected with metabolic disorders [5,6,7]. A prospective Japanese study showed that ambient light exposure was independently associated with subsequent increases in obesity parameters. In particular, increased night-time light exposure corresponded to an estimated 10.2% waist-to-height ratio gain and a 10.0% increase in body mass index (BMI) over 10 years [8]. Type 2 diabetes mellitus, dyslipidaemia, hepatic steatosis, and subclinical carotid atherosclerosis have been associated with ALAN exposure [9,10,11,12]. 

These data suggest that insomnia, exposure to light at night, metabolism, and circadian disruption may start a vicious circle leading to the development of noncommunicable diseases; however, data about the awareness of such a vicious circle in the general population are scarce. 

This data could be a first step toward the knowledge of general awareness, and this simple report could contribute to helping healthcare professionals in finding the way for orienting public policy to sensitise people to the risks of exposure.

Due to the worldwide social and public health importance of these topics, PubMed searching of the related terms retrieves many articles. However, PubMed represents a tool exclusively for scientists and specialists. Google Trends (GT) is helpful in testing widespread knowledge, and has gained interest as a popular means to investigate health topics, being able to provide real-time and archived information on Google queries from 2004 onwards.

## 2. Materials and Methods

Web searches are performed anonymously, and this information can be used to predict human behaviour. Moreover, our study could reveal that people worldwide are unaware about the relationship between health and healthy behaviour, such as avoiding exposure to ALAN during human activities and the relation between diurnal light and nocturnal sleep. Alteration in the circadian rhythm is considered, by the scientific community, as a risk factor for stress, metabolic and sleep disorders, and low performance, particularly in healthcare professionals, towards whom this study is aimed. 

The use of internet data, as an integral part of health informatics, is the base for developing and studying infodemiology. It is a new concept that Eysenbach defined as “the science of distribution and determinants of information in an electronic medium, specifically the Internet, or in a population, with the ultimate aim to inform public health and public policy” [13,14]. 

Thus, also given our previous expertise with this tool [15,16,17,18], considering the complexity of the type of analysis through GT, which we can consider an extremely heterogeneous data source, the aim of the study was to investigate the interest of the general community in the factors of circadian alterations, insomnia, metabolism, and light using GT, analysing the results from different geographical areas and their correlations.

The general population’s research trends on the World Wide Web can be evaluated by GT. This internet resource allows information using appropriate keywords for specific interest areas or periods. GT is a public, accessible, and open-access tool (https://trends.google.com/trends/ accessed on 1 June 2022), and data express patterns and volumes of queries referring to one or more selected search terms [13,14,15,16,17,18,19].

Percentages representing relative search volumes (RSVs) related to a previously defined time frame represent the search results, and are not absolute search volumes. However, they are relative to the other search terms. The output representing seeking activity is proportionately expressed in a data series over a 0 to 100 normalised scale, referred to as a previously selected time. When different search terms are analysed, GT allows comparison RSVs at a global level or sets different geographic areas. As different countries’ populations vary, RSVs are standardised after adjusting the percentage for the population size. 

In order to detect the general population’s interest in the search terms “circadian”, “insomnia”, “metabolism”, and “light”, we compared the RSVs either worldwide or among different areas considering both Anglo-Saxon and European countries.

As the first step, we decided to use the standard conversational English terms “circadian”, “insomnia”, “metabolism”, and “light”; filtering the research, we considered RSVs only by the category “Health” to avoid non-health related results that could interfere the results obtained. We selected the period from 1 January 2007 to 31 December 2021. The entire world and several countries were included in the evaluation, and the geographical position divided the data. Then, we translated the searched terms from English into four different European languages (Italian, French, Spanish, and German) and inserted them into the search string (Table 1).

### Statistical Analysis

We performed a descriptive data analysis to show RSV variations during the study period, comparing different world areas. Mean values of RSVs associated with light, insomnia, metabolism, and circadian in the entire world, United States of America (USA), Australia, United Kingdom (UK), Italy, France, Spain, and Germany were reported, as well as variations of RSV in the same geographical areas. Moreover, we performed a comparison between mean values of relative search volumes associated with light, insomnia, metabolism, and circadian in the entire world, United States of America (USA), Australia, United Kingdom (UK), Italy, France, Spain and Germany, and an evaluation of differences between RSV curves related to search volume associated with circadian, insomnia, light, and metabolism during each month of the study period was carried out; associations between RSVs were tested by calculating Pearson’s correlation after RSV logarithmic transformation. Data analysis was carried out using Microsoft Excel (Microsoft Corp., Albuquerque, NM, USA) and the Statistical Product and Service Solution (SPSS) 26.0 for Windows (IBM Corp., Armonk, NY, USA). A two-sided *p <* 0.05 was considered statistically significant [20].

## 3. Results

**Figure 1:**
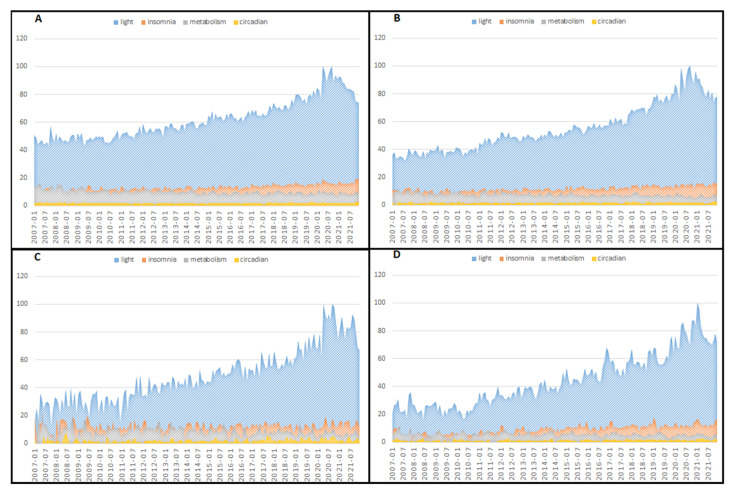
Variation in relative search volume associated with circadian, insomnia, light, and metabolism during each month of the study period for the entire world (**A**), the United States of America (**B**), Australia (**C**), and the United Kingdom (**D**).

**Figure 2:**
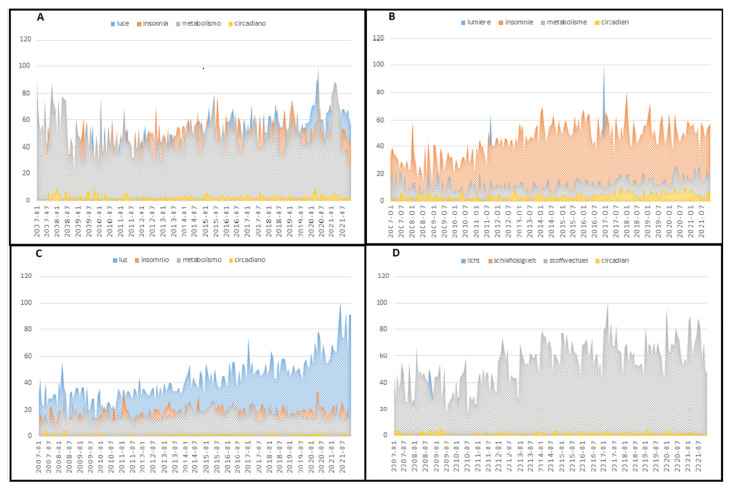
Variation in relative search volume associated with circadian, insomnia, light, and metabolism during each month of the study period for Italy (**A**), France (**B**), Spain (**C**), and Germany (**D**).

The mean RSVs related to the entire world and the single countries analysed during the period considered are reported in Table 2, and variations in RSV during each month are shown in Figure 1 and Figure 2. 

The United States was the country where people searched for light most often, while in Germany and France, the RSVs were lower, suggesting a lower level of interest. It is essential to underline that (as already reported in the Methods section) since the population sizes of each country are different, RSVs are automatically adjusted and standardised on the population. Insomnia had the highest level of searching in Italy and France, while in the USA and Australia, it was lower. Germany and Italy had higher mean RSVs for metabolism, and the USA had lower. Searching for circadian was negligible in all countries analysed. The temporal evaluation showed that searching for light increased from the beginning to the end of the study period in the entire world, in the USA, Australia, and the UK, while searching for insomnia, metabolism, and circadian was stable. A similar temporal pattern was also demonstrated in Spain. At the same time, the other European countries had a temporal searching pattern where metabolism was the general term in Italy and Germany, while insomnia was more frequently searched in France. The strongest correlation between different searching activities was detected between light and insomnia, especially in the USA and the UK. The same relationship was nearly halved in Italy and Spain. Light and metabolism were negatively correlated, especially in the USA, suggesting that populations are unaware. 

The Pearson’s coefficients of the natural logarithm of the relative search volumes associated with circadian, insomnia, light, and metabolism are shown in Table 3.

The highest coefficients were calculated when the natural logarithm of RSV for light and insomnia was analysed; however, such a correlation was lower when data from Australia and Germany were evaluated. A moderate negative correlation was calculated between light and metabolism in the entire world and the USA, while this association was positive in the four European countries. The light was correlated with circadian, having the highest coefficient in France, while correlations of light with circadian and metabolism were weak. Looking at correlation coefficients derived from RSVs associated with global searching, the lowest coefficient was calculated for circadian and light (*r =* 0.169; *p* = 0.023). Moreover, circadian correlated with insomnia (*r =* 0.236; *p* = 0.001) and metabolism (*r =* 0.228; *p* = 0.002). RSVs associated with insomnia correlated strongly with light (*r =* 0.791; *p* < 0.0001) and also mildly with metabolism (*r =* 0.260; *p* < 0.0001). Finally, searching for light was negatively correlated with metabolism (*r =* −0.467; *p* < 0.0001). Evaluation of Anglo-Saxon countries revealed that in the UK, searching for circadian was correlated with insomnia (*r =* 0.306; *p* < 0.0001), light (*r =* 0.360; *p* < 0.0001), and metabolism (*r =* 0.274; *p* < 0.0001). RSV associated with insomnia strongly correlated with light (*r =* 0.767; *p* < 0.0001). In Australia, searching for circadian and insomnia were correlated with light (*r =* 0.359; *p* < 0.0001 and *r =* 0.243; *p* = 0.001, respectively). In the USA, searching for circadian was correlated with light (*r =* 0.257; *p* = 0.001) and weakly with insomnia (*r =* 0.194; *p* = 0.009). Searching for insomnia was strongly correlated with light (*r =* 0.750; *p* < 0.0001) and negatively correlated with metabolism (*r =* -0.232; *p* = 0.002). Finally, light was negatively correlated with metabolism (*r =* −476; *p* < 0.0001). Analysis of European countries showed that in Italy, searching for light was correlated with insomnia (*r =* 0.450; *p* < 0.0001), circadian (*r =* 0.266; *p* < 0.0001), and metabolism (*r =* 0.250; *p* = 0.001). In Spain, searching for metabolism was correlated with insomnia (*r =* 0.312; *p* < 0.0001), light (*r =* 0.271; *p* < 0.0001), and circadian (*r =* 0.223; *p* = 0.003). In the same country, searching for insomnia was correlated with light (*r =* 0.414; *p* < 0.0001). Data related to searching in France showed that circadian was correlated with insomnia (*r =* 0.298; *p* < 0.0001), metabolism (*r =* 0.335; *p* < 0.0001), and light (*r =* 0.402; *p* < 0.0001). Moreover, light was correlated with metabolism (*r =* 0.346; *p* < 0.0001) and insomnia (*r =* 0.586; *p* < 0.0001), and finally, insomnia was correlated with metabolism (*r =* 0.318; *p* < 0.001). In Germany, searching for circadian was correlated with metabolism (*r =* 0.224; *p* = 0.003) and weakly correlated with light (*r =* 0.163; *p* = 0.029). Searching for insomnia was correlated with light (*r =* 0.215; *p* = 0.004) and metabolism (*r =* 0.430; *p* < 0.0001).

## 4. Discussion

With this study, we demonstrate that people worldwide were unaware of the relationship between health and established healthy behaviour, including the circadian rhythm of human activities such as diurnal light and nocturnal sleep. We detected that in different developed countries, such as European countries and the USA, RSVs for exposure to light at night, insomnia, and metabolism had different values, suggesting a dishomogenous level of awareness. Moreover, we detected low awareness regarding the circadian pattern of human activities, and negligible awareness of the possible unhealthy consequence of its disruption. Googling for circadian was meaningless in all evaluated countries. The correlation between light and metabolism was negative in the USA. The health effects of exposure to artificial light seem underestimated; it produces an alteration of the regular daily light profile, thus disrupting the normal circadian sleep–wake cycle and producing circadian desynchronisation. Alteration in the circadian profile is considered a risk factor for stress, metabolic and sleep disorders, and low performance, especially in healthcare professionals [21,22]. 

Our results suggest the potential role of extensive data analysis in understanding the public interest and awareness regarding exposure to light at night, insomnia, altered metabolism, and circadian and disrupted circadian patterns, showing a point of view altogether different from the medical one. These studies should provide information on Internet users’ behaviour/interest in understanding medical language. The internet could act as a form of translator between healthcare professionals and patients. The importance of adapting interventions to population needs, including using languages that patients can understand, should be recognised. GT evaluates the frequency of the users’ web searches; people usually search for information related to an actual problem. Infodemiology is an emerging and exciting area of research that analyses the distribution and circulation of medical information in an electronic medium, able to assess the general population’s consciousness about a specific disease [13,14]. Internet and social media have become very popular, and, for many people around the world, they represent an essential source of information.

Furthermore, using these media, we can investigate and analyse an incredible amount of data originating from people’s searches in real time. Opinions, attention, knowledge, and attitudes can be measured by user-friendly and freely available tools such as GT, and these searches can be limited to health topics. Analysis of the distribution and circulation of public health information in an electronic medium could be aimed at surveillance purposes (info-surveillance) to evaluate the information flows [13,14]. If the general population knows symptoms well, evaluation of search volumes performed using the internet could allow early detection of acute disease outbreaks. Healthcare professionals should be prepared to interpret data derived from internet searching and take advantage of this technology in terms of an increased opportunity to help patients understand and evaluate such information. If this help is lacking, patients’ knowledge cannot improve appropriately through using the internet. Health information reliability is a current problem that healthcare professionals should consider. The internet is now a formidable widespread source of information, with pros and cons, replacing the more traditional scientific forms. Thus, healthcare professionals should not overlook their involvement in such a process.

Data extracted from GT do not represent epidemiological findings, since infodemiology is based on the general population’s knowledge about a given condition. Population knowledge is based on information campaigns; therefore, media coverage can be a significant determinant of search volumes inducing overestimation of benefits, exaggeration of claims, and risk and conflicts of interest may not be disclosed [23]. Different scientific international societies highly recommend global awareness about various health risk factors. Raising awareness about risk factors for different medical conditions encourages systematic screening for dangerous behaviour and stimulates prevention, especially in high-risk populations.

Googling can be considered the first available step to collect information about what people know and understand about a specific disease worldwide. To reiterate, google search refers to looking for information on the World Wide Web. The term suggests looking something up on an internet search engine to include any people or background search to find out anything significant not previously known. Fast-growing internet use by patients to acquire more health information and changes in the health care system towards a patient-centred approach should be considered by clinical researchers.

## 5. Limitations

The authors are aware of the limitation of this study. First, potential anomalies of the data. Several studies [24,25] underline that Google Trends data may be subject to unpredictable fluctuations and anomalies; this aspect was not examined. Second is the mass media effect. Many publications [26,27,28] show that web searches are strongly influenced by mass media coverage, which often misreports scientific evidence. Therefore, caution is required in interpreting the GT results. In the end, no synonyms or common errors in digitation were considered in the selection of keywords. This could influence the causal relationship between the search terms used, and the topic explored.

## 6. Conclusions

Despite the limitations mentioned before and the cautions necessary to use the data, Google has become an essential tool for improving patients’ knowledge. Healthcare professionals should be conscious of these fast changes to help individuals understand and evaluate information [19]. Infodemiology has been accepted as a new science able to assess the distribution and determinants of information in an electronic medium, specifically the internet, or in a population, with the ultimate aim of informing public health and public policy [13,14]. As a more comprehensive application, infodemiology has also become an appreciated and valuable tool for nursing and medical research [15,16,17,18,19], as an easy-to-use source that can describe the general population’s interest in various topics. In particular, GT is an excellent platform for evaluating information-seeking activities and a popular source for big data research. When population knowledge is poor, big data assess the real burden of the knowledge about an unhealthy condition. Our results should be interpreted as a stimulus for researchers in order to use comprehensible language for reaching a wide media coverage. In this way, the scientific community can develop search volumes. The relationship between circadian rhythm, exposure to light at night, insomnia, and altered metabolism leading to low health status must be carefully explained to the general population worldwide.

## Figures and Tables

**Table 1:** Translated searched terms ^1^.

English	Italian	French	Spanish	German
Circadian	Circadiano	Circadien	Circadiano	Circadian
Insomnia	Insonnia	Insomnie	Insomnio	Schlaflosigkeit
Metabolism	Metabolismo	Métabolisme	Metabolismo	Stoffwechsel
Light	Luce	Lumière	Luz	Licht

^1^ Native speaker translated all terms.

**Table 2:** Mean values of relative search volumes associated with light, insomnia, metabolism, and circadian in the entire world, United States of America (USA), Australia, United Kingdom (UK), Italy, France, Spain, and Germany.

	Light	Insomnia	Metabolism	Circadian
**Entire World**	62.2 ± 13.9	13.4 ± 2	10 ± 1.6	2 ± 0.4
**USA**	55.7 ± 16.7	11.7 ± 2	7.7 ± 1.3	1.7 ± 0.5
**Australia**	48.7 ± 19.4	11.9 ± 3.2	8.6 ± 3.5	2 ± 1.4
**UK**	44.8 ± 19	9 ± 3.2	5.6 ± 1.7	1.4 ± 0.6
**Italy**	46.8 ± 16	47.2 ± 11.5	48.8 ± 14.3	2.7 ± 2.1
**France**	27.8 ± 11.9	44.8 ± 12.9	13.1 ± 5.1	3.9 ± 2.5
**Spain**	42.9 ± 15.4	17.5 ± 5	16.5 ± 5	0.8 ± 0.8
**Germany**	28.6 ± 7.4	13.6 ± 5.2	54.7 ± 16.6	1.4 ± 1.3

**Table 3:** Pearson’s coefficients of the natural logarithm of the relative search volume associated with circadian, insomnia, light, and metabolism derived from analysis of the entire world and of the different countries.

	*Circadian*	*Insomnia*	*Light*	*Metabolism*
***Circadian***		Entire world, r = 0.236 ^^^ France, r = 0.298 ^*^ UK, r = 0.306 ^*^ USA, r = 0.194 ^^^	Entire world, r = 0.169 ^+^ France, r = 0.402 ^*^ UK, r = 0.360 ^*^ USA, r = 0.257 ^^^ Australia, r = 0.359 ^*^ Italy, r = 0.266 ^*^ Germany, r = 0.163 ^+^	Entire world, r = 0.228 ^^^ France, r = 0.335 ^*^ UK, r = 0.274 ^*^ Spain, r = 0.223 ^^^ Germany, r = 0.224 ^^^
***Insomnia***	Entire world, r = 0.236 ^^^ France, r = 0.298 ^*^ UK, r = 0.306 ^*^ USA, r = 0.194 ^^^		Entire world, r = 0.791 ^*^ France, r = 0.402 ^*^ UK, r = 0.767 ^*^ USA, r = 0.750 ^*^ Australia, r = 0.243 ^^^ Italy, r = 0.450 ^*^ Germany, r = 0.215 ^^^ Spain, r = 0.414 ^*^	Entire world, r = −0.260 ^*^ France, r = 0.318 ^*^ USA, r = −0.232 ^^^ Germany, r = 0.430 ^*^ Spain, r = 0.312 ^*^
***Light***	Entire world, r = 0.169 ^+^ France, r = 0.402 ^*^ UK, r = 0.360 ^*^ USA, r = 0.257 ^^^ Australia, r = 0.359 ^*^ Italy, r = 0.266 ^*^ Germany, r = 0.163 ^+^	Entire world, r = 0.791 ^*^ France, r = 0.402 ^*^ UK, r = 0.767 ^*^ USA, r = 0.750 ^*^ Australia, r = 0.243 ^^^ Italy, r = 0.450 ^*^ Germany, r = 0.215 ^^^ Spain, r = 0.414 ^*^		Entire world, r = −0.467 ^*^ France, r = 0.346 ^*^ USA, r = −0.476 ^*^ Italy, r = 0.250 ^^^ Germany, r = 0.368 ^*^ Spain, r = 0.271 ^*^
***Metabolism***	Entire world, r = 0.228 ^^^ France, r = 0.335 ^*^ UK, r = 0.274 ^*^ Spain, r = 0.223 ^^^ Germany, r = 0.224 ^^^	Entire world, r = −0.260 ^*^ France, r = 0.318 ^*^ USA, r = −0.232 ^^^ Germany, r = 0.430 ^*^ Spain, r = 0.312 ^*^	Entire world, r = −0.467 ^*^ France, r = 0.346 ^*^ USA, r = −0.476 ^*^ Italy, r = 0.250 ^^^ Germany, r = 0.368 ^*^ Spain, r = 0.271 ^*^	

^*^ p < 0.0001; ^^^ *p* < 0.01; ^+^ *p* < 0.05.

## Data Availability

The datasets generated and/or analysed during the current study are not publicly available, but are available from the corresponding author on reasonable request.
